# Clinical Assessments and EEG Analyses of Encephalopathies Associated With Dynamin-1 Mutation

**DOI:** 10.3389/fphar.2019.01454

**Published:** 2019-12-04

**Authors:** Hua Li, Fang Fang, Manting Xu, Zhimei Liu, Ji Zhou, Xiaohui Wang, Xiaofei Wang, Tongli Han

**Affiliations:** Department of Neurology, Beijing Children’s Hospital, Capital Medical University, National Center for Children's Health, Beijing, China

**Keywords:** epileptic encephalopathy, dynamin-1, mutation, electroencephalogram, children

## Abstract

Epileptic encephalopathy, caused by mutations in the dynamin-1 (*DNM1*; NM_004408) gene, is a newly identified neurologic disorder in children. Thus far, the full clinical and electroencephalographic features of children with *DNM1* mutation-related epileptic encephalopathy have not been established. The aim of this study is to characterize the phenotypic, genetic, and electroencephalographic features of children with *DNM1* mutation-related epileptic encephalopathy. Here, we investigated a patient with a novel pathogenic *DNM1* variant, who received treatment in Beijing Children's Hospital and had detailed clinical, EEG, and genetic information. Conversely, we performed an extensive literature search in PubMed, EMBASE, Cochrane Central Register of Controlled Trials, Chinese BioMedical Literature Database, China National Knowledge Infrastructure, and Wanfang Database using the term “*DNM1*” and were able to find 32 cases reported in nine articles (in English) from January 2013 to December 2018. The clinical features of 33 cases with pathogenic *DNM1* variants were analyzed and the results showed that patients carrying pathogenic variants in the GTPase or middle domains present with epileptic encephalopathy and severe neurodevelopmental symptoms. Patients carrying pathogenic variants in both domains exhibited comparable phenotypes.

## Introduction

Epileptic encephalopathy, caused by dynamin-1 (*DNM1*) mutations, is a newly characterized neurologic disorder in children (Kolnikova et al., 2018). The *DNM1* gene codes for the DNM1 protein is involved in the synaptic vesicle cycle that facilitates the exocytosis of neurotransmitters during receptor-mediated endocytosis, which is necessary for signaling pathway function and central nervous system development. Pathogenic *DNM1* variants affect brain development and function and cause epileptic encephalopathy associated with severe neurodevelopmental complications (Allen et al., 2013; Appenzellar, 2014; Allen et al., 2016; Deng et al., 2016; Nakashima et al., 2016). Previously reported pathogenic variants of *DNM1* have been associated with early onset of epileptic encephalopathy (including West and Lennox-Gastaut syndromes) and are present in up to 2% of patients with infantile spasms or Lennox-Gastaut syndrome (Appenzellar, 2014). For many years, considerable attention has been paid to the genetic studies, patients carrying pathogenic variants in the GTPase or middle domains of *DNM1* exhibit epileptic encephalopathy and severe neurodevelopmental complications. With no clear and effective treatment, antiepileptic medications, as a limited manner, are often insufficient for seizure control in patients with earlier onset and higher frequency of seizures. Thus far, the specific clinical and electroencephalographic features of children with *DNM1* mutation-related epileptic encephalopathy have not been clearly established. Here, we characterized the phenotypic, genetic, and electroencephalographic features of children with *DNM1* mutation-related epileptic encephalopathy.

## Materials and Methods

### Patients

We reported a patient with a novel pathogenic *DNM1* variant, who received treatment in Beijing Children's Hospital and had detailed clinical, EEG and genetic information. In addition, we performed an extensive literature search in PubMed, EMBASE, Cochrane Central Register of Controlled Trials, Chinese BioMedical Literature Database, China National Knowledge Infrastructure and Wanfang Database using the term “*DNM1*,” and were able to find 32 cases reported in nine articles with complete clinical data (in English) from January 2013 to December 2018 (**Table 1**). We then analyzed the clinical features of 33 cases with pathogenic *DNM1* variants, including gender, age at seizure onset, seizure types, development, number of antiepileptic drugs (AEDs) administrated, EEG, and mutations. Seizures and epilepsy syndromes were classified in accordance with the guidelines of the International League Against Epilepsy (Belousova et al., 2017). The analysis of the pathogenic effects of these variations on *DNM1* gene was conducted mainly by using three prediction algorithms: SIFT, PolyPhen 2 Hvar, Mutation taster (**Table 2**).

**Table 1:** Clinical characteristics of patients with pathogenic DNM1 variants.

Case Sequence (reference number)	Sex, age (years)	Seizure onset (months)	Seizure type at onset	Course of seizures	Development	EEG feature
1 (Deng et al., 2016)	M, 5	7	Twitches: Nodded, shrugged, strangled		NVNA; severe ID; hypotonia	SSW, Hyps
2 (Nakashima et al., 2016)	M, 15	11	Neck anteﬂexion, rolling of the eyes, and elevating upper limbs	AtS, MS	NVNA; severe ID; hypotonia	Partial hyps
3 (Nakashima et al., 2016)	M, 6	10	Atonic type with head dropping	AS	NVNA; severe ID; hypotonia	MFED
4 (Appenzellar, 2014)	M, 6	13	ES	Atypical-AS, TS, FS	NVNA; profound ID; hypotonia	Hyps
5 (Appenzellar, 2014)	F, 15	7	ES	Atypical-As with eyelid ﬂuttering, drop attacks, GTCS	Nonverbal; severe ID; hypotonia	Slow bg, MFED
6 (Appenzellar, 2014)	M, 8	6	ES	AtS	NVNA; severe ID; hypotonia	Hyps
7 (Appenzellar, 2014)	F, 13	12	ES	MS, atypical-As, FS, GTCS	NVNA; profound ID; hypotonia	Modified Hyps
8 Appenzellar, (2014)	M, 6	2	ES	SZ-free since age 3.5 years	NVNA; severe ID; hypotonia	High voltage bilateral SSW
9 (Allen et al., 2016)	M, N/A	2	Nodded, Ms	N/A	Profound ID	MFED
10 (von Spiczak et al., 2017)	F, 8	0.75	N/A	AS, MS	NVNA; profound ID; hypotonia	Slow bg
11 (von Spiczak et al., 2017)	M, 2	None	None	None	NVNA; profound ID; hypotonia	Normal
12 (von Spiczak et al., 2017)	M, 8	13	IS	AS, TS, FS, SE	NVNA; profound ID; hypotonia	Hyps, MFED, GPFA, slow bg
13 (von Spiczak et al., 2017)	M, 18	4	IS	AS, TS, GTCS, SE	NVNA; profound ID; hypotonia	Hyps, MFED, SSW, GSW, slow bg
14 (von Spiczak et al., 2017)	F, 15	7	IS	AS, AtS, GTCS	Nonverbal; severe ID; hypotonia	MFED, SSW, slow bg
15 (von Spiczak et al., 2017)	M, 9	2	IS	AS, MS, TS	Nonverbal; severe ID; hypotonia	MFED
16 (von Spiczak et al., 2017)	M, 7	6	IS	AtS, GTCS	Nonverbal; severe ID; hypotonia	Hyps, SSW, FED
17 (von Spiczak et al., 2017)	M, 24	3	IS	AS, GTCS, FS	NVNA; profound ID; hypotonia	MFED, SSW, slow bg
18 (von Spiczak et al., 2017)	M, 3	5	IS	AS, AtS, GTCS	NVNA; profound ID; hypotonia	Hyps, MFED, GSW, slow bg
19 (von Spiczak et al., 2017)	M, 2 (sib of 21)	4	IS	MS, GTCS, FS	NVNA; profound ID; hypotonia	Hyps, MFED, FED, slow bg
20 (von Spiczak et al., 2017)	F, 5 (sib of 20)	N/A	IS	MS, TS, GTCS, FS	NVNA; profound ID; hypotonia	MFED, FED, slow bg
21 (von Spiczak et al., 2017)	M, 12	5	IS	AS, MS, AtS, GTCS	NVNA; profound ID; hypotonia	Hyps, MFED, SSW, GSW, GPFA, slow bg
22 (von Spiczak et al., 2017)	M, 19	8	N/A	GTCS	NVNA; profound ID; hypotonia	N/A
23 (von Spiczak et al., 2017)	M, 13	6	IS	MS, TS	Nonverbal; profound ID; hypotonia	Hyps, MFED, FED, slow bg
24 (von Spiczak et al., 2017)	M, 1	3	None	None	NVNA; profound ID; hypotonia	MFED, slow bg
25 (von Spiczak et al., 2017)	F, 1	1	IS	MS, TS, GTCS, FS, SE	NVNA; profound ID; hypotonia	Hyps, MFED, SSW, GSW, GPFA
26 (von Spiczak et al., 2017)	F, 5	4.5 years	N/A	MS, TS, GTCS, FS, rSE	NVNA; profound ID	GSW, slow bg
27 (von Spiczak et al., 2017)	M, 2	3	IS	MS	NVNA; hypotonia	Hyps, MFED, slow bg
28 (Kolnikova et al., 2018)	F, 8	1 day	MS, TS	IS, MS	Nonverbal, unable to roll over or sit alone, profound ID	Abnormal bg, SSW over right hemisphere;
29 (Allen et al., 2013)	N/A	Infancy	N/A	N/A	N/A	N/A
30 (Lazzara et al., 2018)	M, 12	5 days	Neonatal seizures	FS, CE, SE	N/A; severe ID; hypotonia	Mild slow bg and MFED
31 (Brereton et al., 2018)	F, 8	N/A	N/A	N/A	Sit and walk without support; minimal speech; mild-moderate ID; hypotonia	Normal
32 (Brereton et al., 2018)	F, 8	N/A	N/A	Age 5, an episode of unresponsiveness and eye deviation to the left side, characterized by back arching and leaning to the left side	Sit and walk without support; limited speech; mild-moderate ID; hypotonia	Normal
The present case	F, 0.6	8	IS	IS	NVNA; severe ID; hypotonia	Sharp wave, MFED

DNM1, dynamin-1; F, female; M, male; DD, developmental delay; N/A, not available or applicable; IS, infantile spasms; ES, epileptic spasms; AS, absence seizures; Ats, atonic seizures; FS, focal seizures; TS, tonic seizures; GTCS, generalized tonic-clonic seizures; CE, convulsive seizures; SE, status epilepticus; MS, myoclonic seizure; SZ, seizure; CLB, clobazam; VGB, vigabatrin; VPA, valproic acid; Hyps, hypsarrhythmia; KD, ketogenic diet; bg, background; GPFA, generalized paroxysmal fast activity; GSW, generalized spike-wave or poly spike-wave discharges; MFED, multifocal epileptiform discharges; FED, focal discharges; SSW, slow spike-wave discharges; CC, corpus callosum; MCM, mega cisterna magna; ID, intellectual disability; FL, frontal lobe; NVNA, nonverbal, non-ambulatory.

**Table 2:** Details of the DNM1 mutations variants of 33 cases.

Case Sequence (reference number)	Domain involved	Mutation	MutationTaster	Polyphen-2	SIFT	SIFT SCORE	rs. number
1 (Deng et al., 2016)	GTPase	c.443A > G (p.Gln148Arg)	Disease causing	Probably damaging	Damaging	0	NO rs
2 (Nakashima et al., 2016)	GTPase	c.127G > A (p.Gly43Ser)	Disease causing	Probably damaging	Damaging	0.001	NO rs
3 (Nakashima et al., 2016)	GTPase	c.709C > T (p.Arg237Trp)	Disease causing	Probably damaging	Damaging	0	rs760270633
4 (Appenzellar, 2014)	GTPase	c.194C > A (p.Thr65Asn)	Disease causing	Probably damaging	Damaging	0	NO rs
5 (Appenzellar, 2014)	GTPase	c.529G > C (p.Ala177Pro)	Disease causing	Probably damaging	Damaging	0.001	rs587777860
6 (Appenzellar, 2014)	GTPase	c.618G > C (p.Lys206Asn)	Disease causing	Probably damaging	Damaging	0	rs587777861
7 (Appenzellar, 2014)	GTPase	c.709C > T (p.Arg237Trp)	Disease causing	Probably damaging	Damaging	0	rs76020633
8 (Appenzellar, 2014)	Middle	c.1076G > C (p.Gly359Ala)	Disease causing	Probably damaging	Damaging	0.001	rs587777862
9 (Allen et al., 2016)	GTPase	c.865A > T (p.Ile289Phe)	Disease causing	Probably damaging	Damaging	0.01	NO rs
10 (von Spiczak et al., 2017)	GTPase	c.127G > A (p.Gly43Ser)	Disease causing	Probably damaging	Damaging	0	NO rs
11 (von Spiczak et al., 2017)	GTPase	c.134G > A (p.Ser45Asn)	Disease causing	Probably damaging	Damaging	0	NO rs
12 (von Spiczak et al., 2017)	GTPase	c.194C > A (p.Thr65Asn)	Disease causing	Probably damaging	Damaging	0	NO rs
13 (von Spiczak et al., 2017)	GTPase	c.416G > T (p.Gly139Val)	Disease causing	Probably damaging	Damaging	0	NO rs
14 (von Spiczak et al., 2017)	GTPase	c.529G > C (p.Ala177Pro)	Disease causing	Probably damaging	Damaging	0.001	rs587777860
15 (von Spiczak et al., 2017)	GTPase	c.616A > G (p.Lys206Glu)	Disease causing	Probably damaging	Damaging	0	NO rs
16 (von Spiczak et al., 2017)	GTPase	c.709C > T (p.Arg237Trp)	Disease causing	Probably damaging	Damaging	0	rs760270633
17 (von Spiczak et al., 2017)	GTPase	c.709C > T (p.Arg237Trp)	Disease causing	Probably damaging	Damaging	0	rs760270633
18 (von Spiczak et al., 2017)	GTPase	c.709C > T (p.Arg237Trp)	Disease causing	Probably damaging	Damaging	0	rs760270633
19 (von Spiczak et al., 2017)	GTPase	c.709C > T (p.Arg237Trp)	Disease causing	Probably damaging	Damaging	0	rs760270633
20 (von Spiczak et al., 2017)	GTPase	c.709C > T (p.Arg237Trp)	Disease causing	Probably damaging	Damaging	0	rs760270633
21 (von Spiczak et al., 2017)	GTPase	c.709C > T (p.Arg237Trp)	Disease causing	Probably damaging	Damaging	0	rs760270633
22 (von Spiczak et al., 2017)	GTPase	c.713G > A * (p.Ser238Ile)	N/A	N/A	Damaging	0	NO rs
23 (von Spiczak et al., 2017)	Middle	c.1037G > T (p.Gly346Val)	Disease causing	Probably damaging	Damaging	0	rs1064794903
24 (von Spiczak et al., 2017)	Middle	c.1075G > A (p.Gly359Arg)	Disease causing	Probably damaging	Damaging	0	NO rs
25 (von Spiczak et al., 2017)	Middle	c.1075G > A (p.Gly359Arg)	Disease causing	Probably damaging	Damaging	0	NO rs
26 (von Spiczak et al., 2017)	Middle	c.1117G > A (p.Glu373Lys)	Disease causing	Probably damaging	Damaging	0.046	NO rs
27 (von Spiczak et al., 2017)	Middle	c.1190G > A (p.Gly397Asp)	Disease causing	Probably damaging	Damaging	0.002	NO rs
28 (Kolnikova et al., 2018)	Middle	c.1089_1090insCTTCCA (p.Asn363_Arg364insLeuPro)	polymorphism	N/A	N/A		NO rs
29 (Allen et al., 2013)	Middle	c.1190G > A (p.Gly397Asp)	Disease causing	Probably damaging	Damaging	0.002	NO rs
30 (Lazzara et al., 2018)	Middle	c.796C > T (p. Arg266Cys)	Disease causing	Probably damaging	Damaging	0	rs138053929
31 (Brereton et al., 2018)	PH	c.1603A > G (p.Lys535Glu)	Disease causing	possibly damaging	Damaging	0.002	NO rs
32 (Brereton et al., 2018)	PH	c.1603A > G (p.Lys535Glu)	Disease causing	possibly damaging	Damaging	0.002	NO rs
Present case	GTPase	c.135C > A (p.Ser45Arg)	Disease causing	Probably damaging	Damaging	0.006	NO rs

Transcript ID: ENST00000372923. *Transcription is unknown. N/A, not available.

### Statistical Analysis

The continuous variables were described by mean or median with range, and the categorical variables by number or percentage. Chi-square tests were used for comparison of phenotypic difference with mutations in different gene domains. *P*-value below 0.05 was considered signiﬁcant. Statistical analysis was performed using SPSS 22.0.

### Ethics Statement

The present study was approved by the Ethics Committee of Beijing Children's Hospital and informed consents were collected from the participant's parents.

## Results

### Case Report

**Figure 1:**
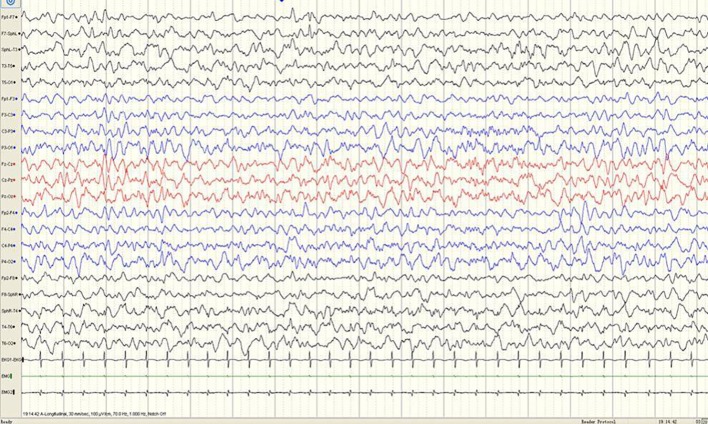
A sample EEG recording of 4-month-old child in this study. The interictal EEG recording shows a sharp slow wave discharge in the left anterior temporal region.

**Figure 2:**
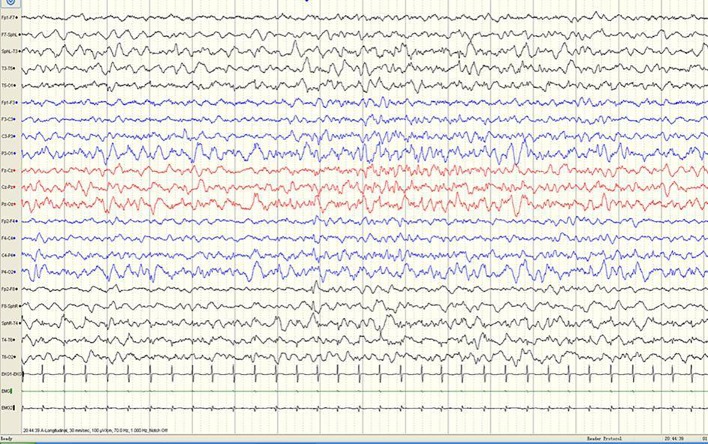
A sample EEG recording of 4-month-old child in this study. The interictal EEG recording shows a sharp slow wave discharge in the right anterior temporal region.

**Figure 3:**
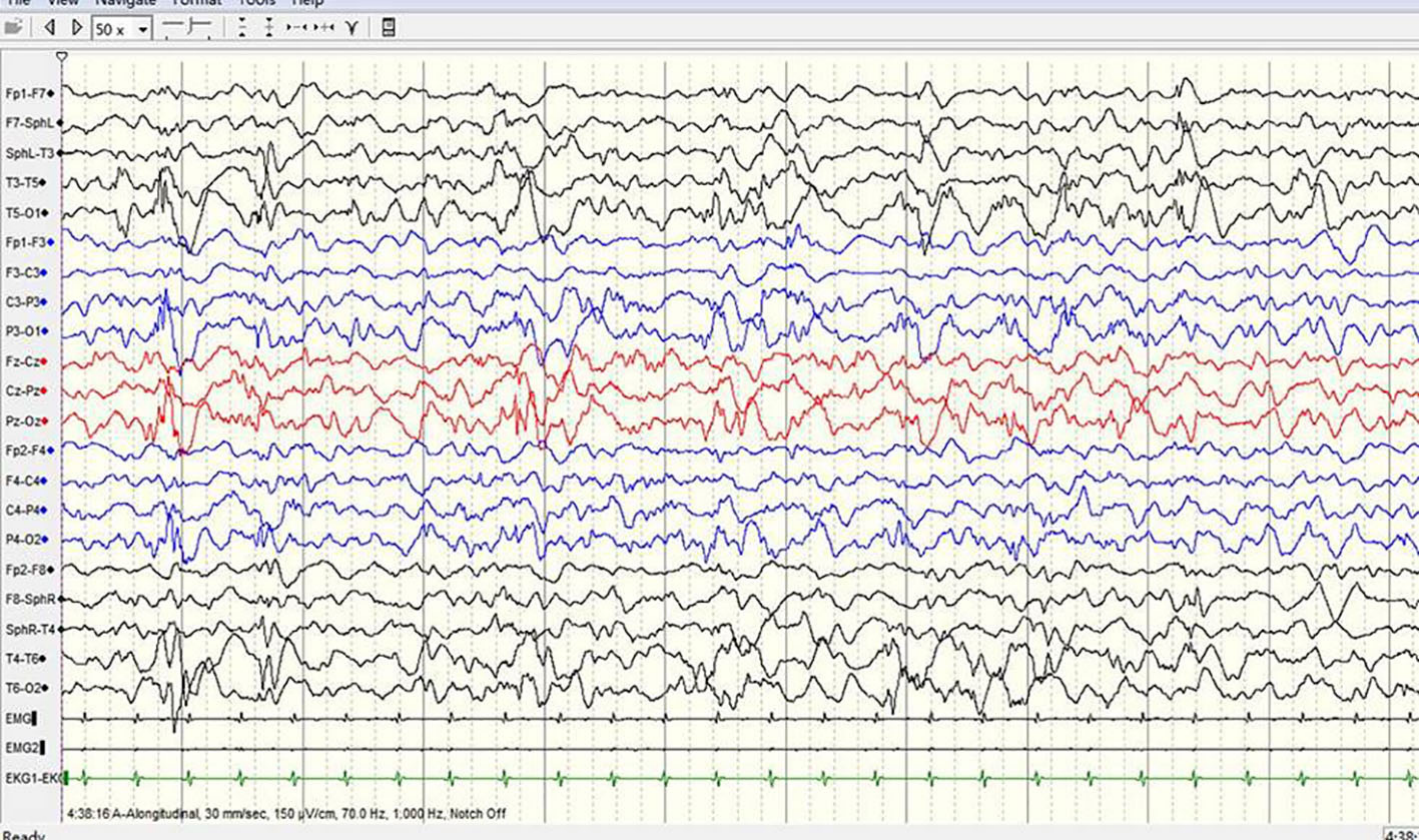
A sample EEG recording of 8-month-old child in this study. The interictal EEG recording shows prime spike waves, a small number of multiple spike waves, spike-slow waves, and multiple spike-slow wave synchronous or non-synchronous discharge (atypical hypsarrhythmia) onto the bilateral rear head.

**Figure 4:**
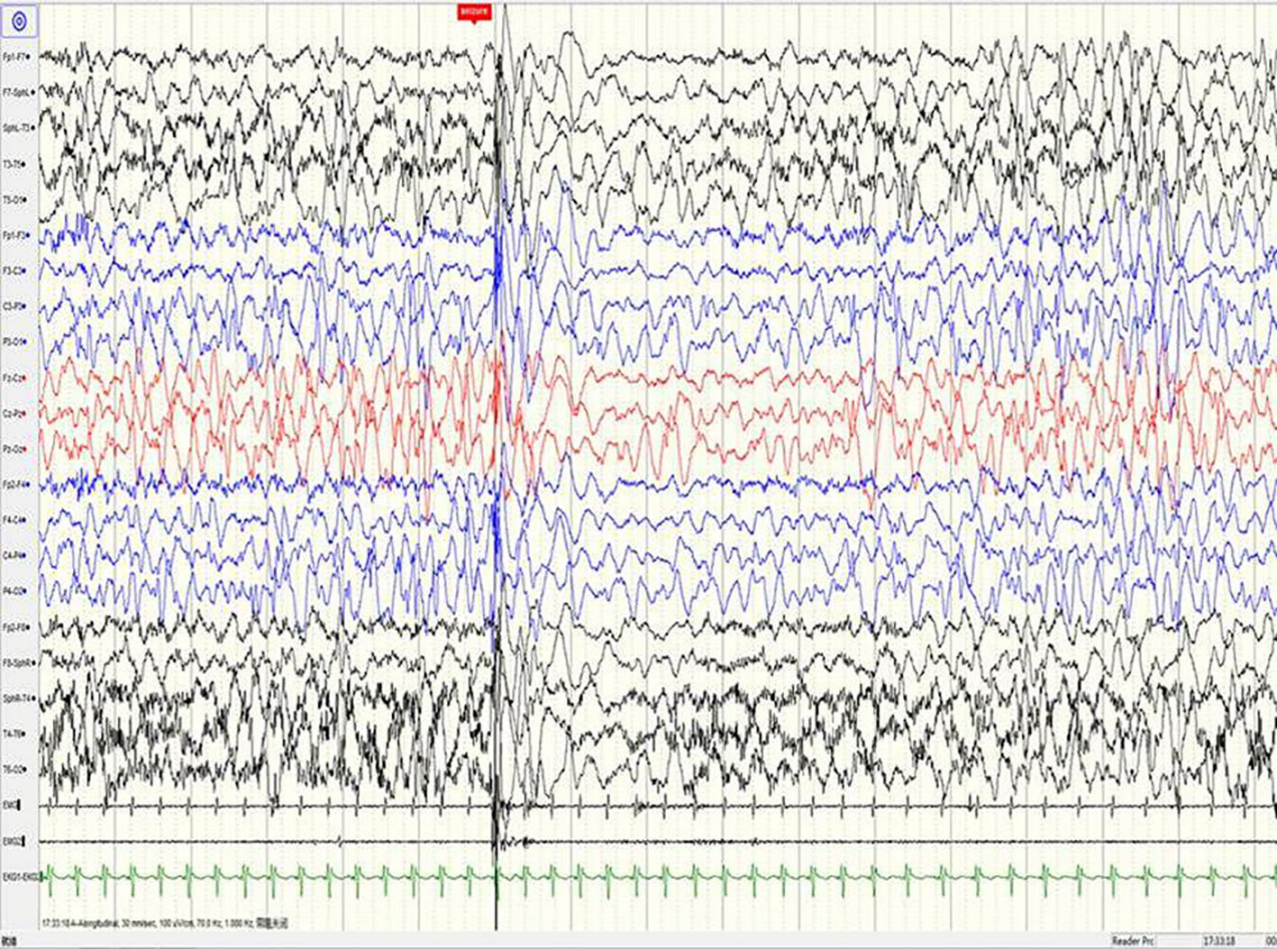
A sample EEG recording of 8-month-old child in this study. The seizures are characterized by left oblique movement of both eyes and limb flexion uplift; the same period of the EEG signals show bilateral lead extensive spike-slow wave and myoelectric outbreak in the bilateral deltoid muscle.

A 3-year-old girl presented with severe psychomotor developmental delay; nonverbal and non-ambulatory. She had been delivered at full term after 40 weeks of gestation (birth weight 3,150 g, Apgar score 10), and was the first child of the family. The patient has normal personal and family history. She exhibited limb shaking at 2 months after birth and EEG analysis revealed no epileptiform discharges. Over the following 4 months, she exhibited shaking limbs intermittently upon waking. At 6 months of age, the patient exhibited “binocular vision and tongue vomiting” which gradually increased over time, although the time of onset was unclear. Video-EEG monitoring was performed multiple times, based on the suspicion of non-epileptic seizures. Levetiracetam was administrated during observation. Infantile spasms manifested at 8 months after birth. EEG analysis showed prime spike waves, a small number of multiple spike waves, spike-slow waves, and synchronous or non-synchronous discharge (atypical hypsarrhythmia) onto the bilateral rear head during seizures. Subsequent treatment (beginning at 8 months of age) constituted administration of levetiracetam and topiramate. Changes in EEG are shown in **Figures 1**–**4**. The bilateral ventricles were slightly widened on magnetic resonance images beginning at 2 months of age.

**Figure 5:**
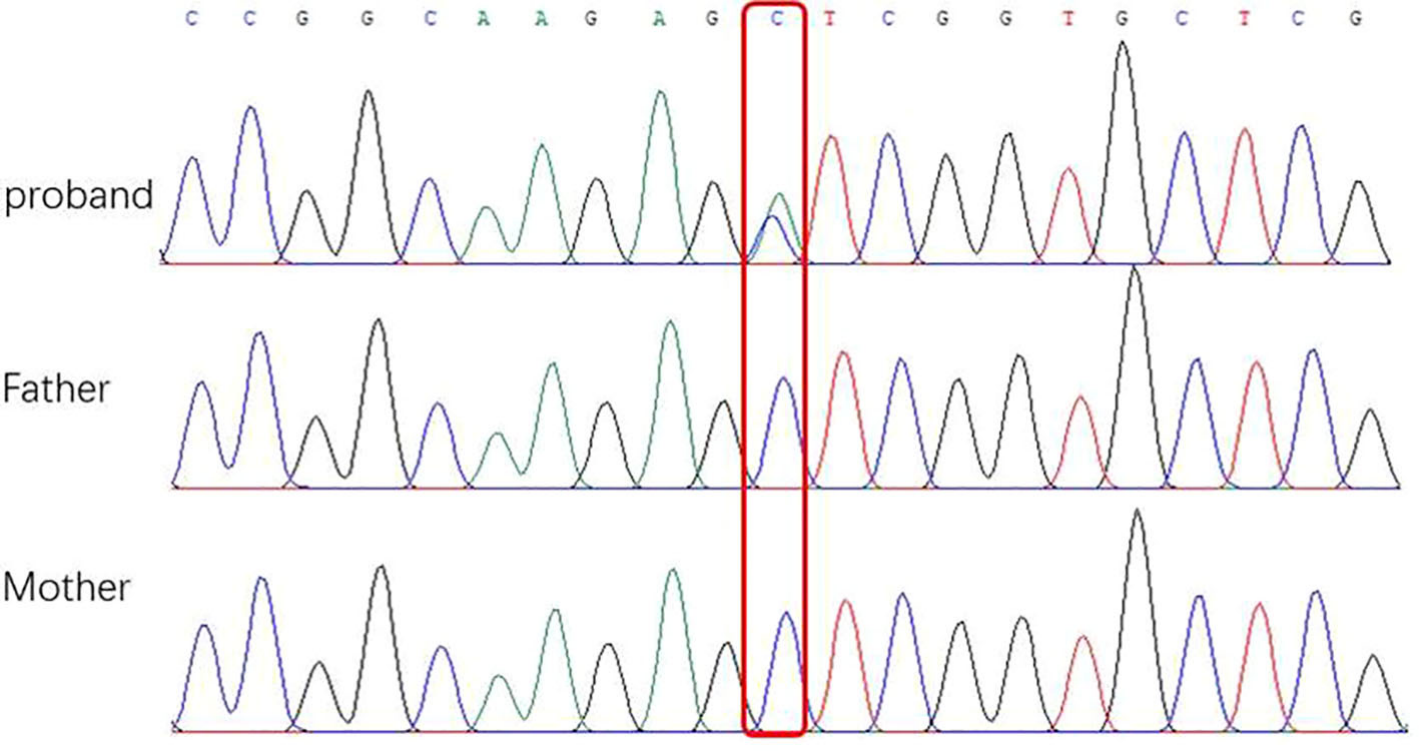
Sequencing of *DNM1* gene mutations of children and their parents in this study. **(A)** Base 135 of the coding sequence of the DNA of a patient in this group shows a missense mutation c.135C > A (p.GluS45R) (arrow). **(B)** and **(C)** are the corresponding gene sites in the father and mother, respectively; these sites (arrow) do not show the mutation.

**Figure 6:**
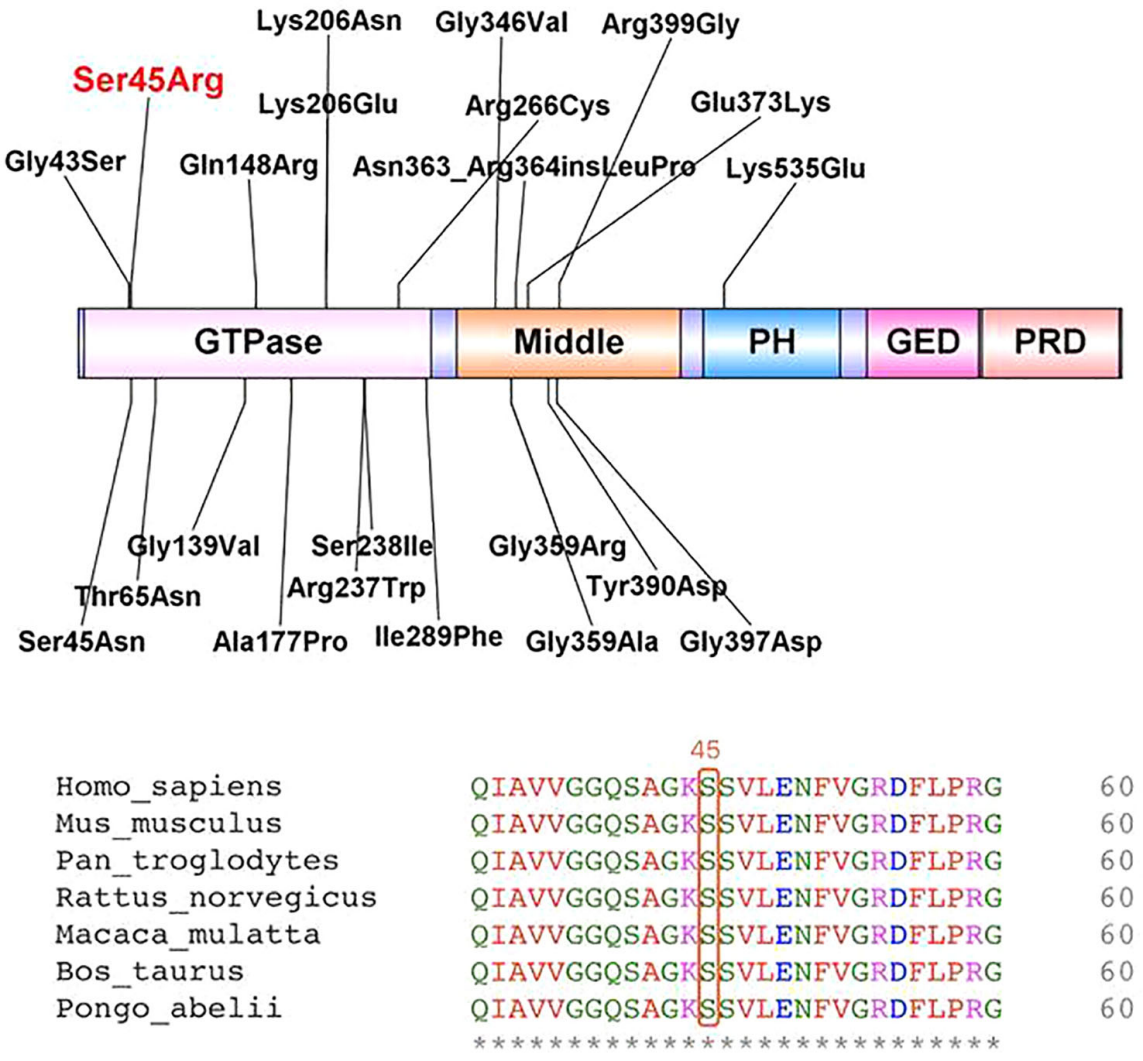
Locations of identified variants in dynamin-1 protein structure (top) and amino acid sequence of the present variant (bottom).

After obtaining informed consent from the parents, whole exome sequencing (WES) of the patient and parental samples were analyzed by a trio-based analysis, which identified a variant, c.135C > A, in the *DNM1* gene (NM_004408), with the amino acid changes of p.Ser45Arg (**Figure 5**), which was confirmed by Sanger sequencing. To the best of our knowledge, this was an unreported *de novo* mutation. This missense mutation is absent in gnomAD, ExAC, 1000 Genomes, and ESP 6500 databases; moreover, it is predicted to be a disease variant by Polyphen-2 (score of 0.988), MutationTaster (disease causing), SIFT (score of 0.006), and M-CAP (score of 0.979). According to sequence alignment, the Ser45 residue is highly conserved across species, indicating evolutionary importance (**Figure 6**).

### Clinical Characteristics of Patients With Pathogenic *DNM1* Variants

Our data showed that patients carrying pathogenic variants in the GTPase or middle domains present with epileptic encephalopathy and severe neurodevelopmental symptoms. For the analysis of *DNM1*-related encephalopathy, 31 out of the 33 patients were included (9 females, 21 males, the sex of one patient was not available). The age range of the patients was 0.6–24 years, the median age was, at inclusion, 8 years. Pregnancy and delivery were unremarkable in all patients with normal birth parameters. Patient 25 died at 2 years of age, before enrolment in this study. The clinical characteristics of *DNM1* mutation-related epileptic encephalopathy patients were analyzed as follows:

#### Seizures

From the 31 patients with *DNM1* mutation-related epileptic encephalopathy, 29 (93.5%) experienced epileptic seizures. Patient 11 did not have seizures, and patient 24 showed only subcortical, nonepileptic myoclonic jerks. Seizures began at a median age of 5 months (range 1 day to 4.5 years). Patient 26 was an outlier with onset at 4.5 years with a febrile infection related epilepsy syndrome phenotype. During the course of the disease, 23 (74.2%) patients had spasm seizures, 12 (38.7%) patients had absence seizures, 9 (29.0%) patients had tonic seizures, 12 (38.7%) patients had myoclonic seizures, 5 (16.1%) patients had atonic seizures, 13 (41.9%) patients had generalized tonic-clonic seizures, and 9 (29.0%) patients had focal seizures (**Table 1**). Fourteen out of 15 patients (93.3%) presented with infantile spasms initially, whereas 1 patient presented with myoclonic seizures, tonic seizures, generalized tonic-clonic seizures (GTCS), and focal seizures. Information was not available for one patient.

#### Development

All patients with *DNM1* mutation-related epileptic encephalopathy were nonverbal except for two patients, which were not mentioned in literature, with severe to profound intellectual disability. In 24 out of the 31 patients (77.4%), the developmental delay was apparent before seizure onset. Except for six patients who had normal development until the onset of refractory seizures, all patients had considerable developmental delays in the first year of life. Twenty-eight out of the 31 patients (90.3%) were non-ambulatory.

#### Response to Treatment

Seizure outcome was assessed in 31 patients: 24 out of 31 patients (77.4%) had refractory seizures. Three patients (9.7%) became seizure-free post treatment. Patient 8 became seizure-free after placement on ketogenic diet at the age of 3.5 years while patient 6 had some response to ketogenic diet. Seizures in patients 1, 2, and 5 were controlled with valproic acid, clobazam, or vigabatrin over a period of 5 years between the ages 3 to 8 years.

In addition, patients 31 and 32, carrying pathogenic variants in the pleckstrin homology domain exhibited milder phenotypes without epilepsy. The two girls, 8 years old, were monozygotic triplet sisters who presented for evaluation of developmental delay, autism spectrum disorder, some dysmorphic features, and hypotonia without repeated seizures.

#### EEG Results

The EEG results of patients 31 and 32 were normal. EEG results were abnormal in 30 out of 31 (96.7%) patients with *DNM1* mutation-related epileptic encephalopathy. The EEG patterns of five patients reveal varied epileptiform discharges initiating as hypsarrhythmia, and evolving from slow generalized spike-wave discharges to paroxysmal fast activity. One patient exhibited non-specific background activity. Among the 31 patients, 15 (48.4%) had epileptiform discharge and background slowing. Twenty-eight (90.3%) patients had epileptiform discharge, of which multifocal discharge was the most common. There were 18 (58.1%) patients with multifocal discharge and 14 (45.2%) patients with hypsarrhythmia (**Figure 3**). Regarding other epileptiform discharges, there were nine (29.0%) patients with slow-spike wave complex, three (9.7%) patients with fast-wave activity, three (9.7%) patients with extensive spike activity, and four (12.9%) patients with focal epileptiform discharge. In addition, there were two (6.5%) patients with multifocal discharge or hypsarrhythmia and slow-spike wave complex.

#### DNM1 Mutation Results

The current study reviewed data from 33 patients, including 31 sporadic patients and a sibling pair (patients 20 and 21), resulting in a total of 20 independent mutations (**Table 2**). The most common mutation was c.709C.T (p.Arg237Trp), which was found in 8 out of the 33 independent patients (24.2%). All mutations were confirmed to be *de novo*, except for the affected sibling pair. Twelve of 20 mutations (60.0%), including the recurrent c.709C.T (p.Arg237Trp) mutation, occurred in the GTPase domain of *DNM1*.Seven out of the 20 mutations (35.0%) occurred in the middle domain of *DNM1*. One (5.0%) occurred in the PH domain of *DNM1*. Twenty novel missense/frame-insertion mutations were predicted as pathogenic using the *in silico* prediction tools Mutation Taster Server, Polyphen-2, and SIFT.

#### Comparison of Genetic and Clinical Phenotypes of Children With DNM1 Mutation-Related Encephalopathy

As shown in **Table 3**, sex (female vs. male, *P* = 0.5935), age at seizure onset (< 6 months vs. > 12 months vs. 6–12 months, *P* = 0.4007), seizure type at onset (infantile/epileptic spasms vs. other type, *P* = 0.5491), seizure outcome (intractable vs. seizure-free, *P* = 0.1145), and intellectual disability (profound vs. severe, *P* = 0.5523) showed no significant associations with the GTPase or middle domains.

**Table 3:** Comparison of gene domains and clinical features of 31 cases with mutation-related epileptic encephalopathy.

	Domain involved				χ^2^	*P*
	GTPase		Middle			
	No.	Percent	No.	Percent		
**Sex**					0.285	0.5935
Female	6	27.27%	3	37.50%		
Male	16	72.73%	5	62.50%		
**Age at seizure onset**					1.829	0.4007
<6 months	9	42.86%	6	66.67%		
>12 months	2	9.52%	1	11.11%		
6–12 months	10	47.62%	2	22.22%		
**Seizure type at onset**					0.359	0.5491
IS/ES	15	78.95%	4	66.67%		
Other type	4	21.05%	2	33.33%		
**Seizure outcome**					—	0.1145
Intractable	20	95.24%	4	66.67%		
Seizure-free	1	4.76%	2	33.33%		
**ID**					0.353	0.5523
Profound	13	59.09%	5	71.43%		
Severe	9	40.91%	2	28.57%		

IS, infantile spasms; ES, epileptic spasms; ID, intellectual disability.

## Discussion

Neurotransmission in the central system relies on synaptic vesicle transport. DNM1 is a protein involved in the synaptic vesicle cycle, which facilitates the exocytosis of neurotransmitters necessary for normal signaling pathways and development in the central nervous system. Dynamin proteins have five domains; the GTPase domain is the largest and best understood, followed by a middle domain, a pleckstrin homology domain, a GTPase effector domain, and a proline-rich domain (McNiven et al., 2000). The pleckstrin homology domain is thought to interact directly with the lipid bilayer. The *DNM1* gene is mainly expressed in the central nervous system (Romeu and Arola, 2014), which explains the neurological phenotypes in *DNM1*-related disorders. Next-generation sequencing has been rapidly implemented into routine clinical practice, where it has improved the diagnostic rate of patients with neuromuscular diseases. The widespread application of next-generation sequencing has greatly facilitated the understanding of the underlying mechanisms of epileptic encephalopathy (Fang et al., 2017; Ni and Shi, 2017). Previous publications have characterized the functional consequences of *DNM1* mutations and found that the seizure phenotype is largely due to the deleterious effects of *DNM1* mutations in GABAergic interneurons, while behavioral locomotor phenotypes may be due to the effect of the mutation in pyramidal cells (Asinof et al., 2015; Asinof et al., 2016).

Based on the collected mutation pattern and clinical information, we analyzed the relationship between genotypes and phenotypes. Previous research interpreted the molecular mechanisms of *DNM1* mutations and inferred the connection between genotypes and phenotypes to certain extent. It has been reported that mutations in different domains lead to distinct clinical phenotypes. Patients carrying pathogenic variants in the GTPase or middle domains present with epileptic encephalopathy and severe neurodevelopmental symptoms. These mutations have been reported in association with early onset epileptic encephalopathy (Appenzellar, 2014), intractable seizures (seizure onset in *DNM1* patients ranges from 2–13 months of and usually presents with infantile spasms. The seizure type manifests in various forms as the patient ages, ranging from absence seizures to generalized tonic-clonic seizures.), motor impairments, and severe to profound intellectual disability. In this study, 24 patients (77.4%) had refractory seizures. During the course of the disease, 23 (74.2%) patients initially had spasm seizures; all patients had severe to profound intellectual disability and considerable developmental delay in the first year of life. Other clinical features reported in some affected individuals included hypotonia, developmental regression, movement disorder, autism, cortical visual impairment, behavioral concerns, and microcephaly. Patients carrying pathogenic variants in both domains exhibited comparable phenotypes (**Table 3**), although the mechanism of protein disruption was distinct from that of patients with variants in a single domain. Most variants in the GTPase domain were predicted to impair hydrolysis of GTP, but not its binding to the synaptic vesicle; this resulted in integrated oligomeric assembly and impaired vesicle scission. However, middle domain variants were predicted to impair the ability of the DNM1 protein to form larger oligomeric assemblies. In the case of the patients, the dominant negative effect of *DNM1* results in a generally similar overall phenotype suggesting a similar pathway (von Spiczak et al., 2017). Patients carrying pathogenic variants in the pleckstrin homology domain exhibited milder phenotypes without epilepsy. These patients were 8-year-old identical twin sisters who had no seizures and exhibited mild-to-moderate developmental delay/intellectual disability and autism spectrum disorder (Brereton et al., 2018). The *de novo* p.Lys535Glu mutation is a likely pathogenic novel variant in exon 15 of *DNM1*. However, this was reported in a single patient without an epilepsy phenotype. Therefore, reports of additional patients are needed to define the relationship between genotype and phenotype in *DNM1* mutation-related epileptic encephalopathy (Jia and Shi, 2017).

EEG is an important tool for assessment of the diagnosis and prognosis of epileptic encephalopathy in patients carrying *DNM1* mutations. The EEG patterns are consistent with changes in the electrical activities of the brain in patients with infantile epileptic encephalopathy. The EEG patterns reveal varied epileptiform discharges initiating as hypsarrhythmia and evolving from slow generalized spike-wave discharges to paroxysmal fast activity. In this study of epileptic encephalopathies in 31 patients carrying *DNM1* mutations (**Table 1**), approximately 96.7% of patients' recordings portrayed abnormal EEG; multifocal discharge was most common (58.1%), followed by hypsarrhythmia (45.2%). Other epileptiform discharges were characterized by slow-spike and slow-wave complex, fast-wave activity, extensive spike activity, and focal epileptic discharge. The results of this study were consistent with those of a retrospective study published in 2017. The specific EEG pattern remains the basis for the diagnosis. Series EEG with video and video EEG with electromyogram electrodes is also recommended. The association of characteristic multiple seizure types and intellectual disability represents the classic hallmark of Lennox-Gastaut syndrome. This diagnostic triad may not be completely present at the onset of seizures; therefore, an accurate diagnosis of Lennox-Gastaut syndrome often requires further disease development over time (Markand, 2003; Arzimanoglou et al., 2009; Camfield, 2011; Bourgeois et al., 2014). The patient in the present case underwent a series of video EEG monitoring, which initially showed focal discharge, followed by atypical hypsarrhythmia and infantile spasms; this suggested evolution of the disease and provided clues for diagnosis and treatment.

The long-term outcomes of patients with *DNM1* mutation-related epileptic encephalopathy were often disappointing. The choice of AEDs at the onset of seizures was tailored to seizure type, clinical presentation, and EEG pattern. Thus far, there are no international guidelines for the pharmacological treatment of *DNM1* mutation-related epileptic encephalopathy because of the limited efficacy of antiepileptic medications. In the present study, three patients had been given sodium valproate and became seizure-free; however, as the disease progressed, they developed drug-refractory epilepsy. Therefore, we analyzed differences in the response of the same gene mutation to drug treatment. First, we speculated that the choice of treatment time or the natural process of disease might influence the response. Then, we investigated whether the type and site distributions of *DNM1* gene mutations were associated with clinical phenotype, potentially providing clues for clinical diagnosis and treatment. Eight patients carried the p.Arg237Trp mutation (**Tables 1** and **2**). Given that *DNM1* mutations are present in up to 2% of patients with severe epilepsy (Kolnikova et al., 2018), this mutation is particularly frequent in patients with epileptic encephalopathy. The relatively homogeneous phenotype and predicted dominant-negative mechanism of this mutation make *DNM1*-associated encephalopathy has the potential of being an effective therapeutic target. Gene therapy might also be an effective means to restore *DNM1* function (Kolnikova et al., 2018). We presume that treatment methods and strategies will be further refined with additional studies involving more patients and investigations into the molecular basis of the disease.

In conclusion, to the best of our knowledge, this is the first integrated analysis of the phenotypic, genetic, and electroencephalographic features of children with *DNM1* mutation-related encephalopathy. Our study highlighted the role of series EEG and video EEG of children with *DNM1* mutation-related encephalopathy; EEG patterns may aid in providing clues for treatment.

There were several limitations to this study, such as the fact that it is a retrospective and summary study. Due to the small number of cases, we have not come to a definite conclusion; the pathogenic variant in this study needs to be confirmed by functional experiments. To determine the association of phenotype and genotype of children with *DNM1* mutation-related encephalopathy, further analysis of additional patients is needed.

## Ethics Statement

The present study was approved by the Ethics Committee of Beijing Children's Hospital and the patient have gave written informed consent.

## Author Contributions

All authors contributed to the study design, critically reviewed the manuscript, and approved the final version. HL performed literature search and analysis, and wrote the manuscript. MX, ZL, JZ, XiaohW, XiaofW, and TH performed literature search and analysis. FF revised the manuscript.

## Funding

This work was supported by National Natural Science Foundation of China(81541115), the Capital Health Research and Development Fund(2018-2-2096) and Beijing Municipal Administration of Hospitals Incubating Program(PX2017065).

## Conflict of Interest

The authors declare that the research was conducted in the absence of any commercial or financial relationships that could be construed as a potential conflict of interest.
